# High-Throughput Screening of Nanoparticle-Stabilizing Ligands: Application to Preparing Antimicrobial Curcumin Nanoparticles by Antisolvent Precipitation

**DOI:** 10.1007/s40820-014-0020-6

**Published:** 2014-11-22

**Authors:** Ilya Shlar, Elena Poverenov, Yakov Vinokur, Batia Horev, Samir Droby, Victor Rodov

**Affiliations:** 1 Institute of Postharvest and Food Sciences, Agricultural Research Organization, The Volcani Center, Bet Dagan, Israel; 2 Institute of Biochemistry, Food Science and Nutrition, Hebrew University of Jerusalem, Rehovot, Israel

**Keywords:** Nanoparticles, Curcumin, Antisolvent precipitation, Stabilizing ligands, High-throughput screening, Antimicrobial, *E. coli*

## Abstract

Water-dispersible curcumin nanoparticles were prepared by bottom-up antisolvent precipitation approach. A new high-throughput screening technique was developed for selecting appropriate ligands stabilizing the nanoparticles in aqueous medium and improving their performance. The initial set of twenty-eight potential stabilizing ligands was evaluated based on their capacity to improve curcumin dispersibility in aqueous medium. The performance of four promising ligands (amino acid proline, polyphenol tannic acid, polycation Polyquaternium 10, and neutral polymer polyvinylpyrrolidone) was tested in ultrasound-aided antisolvent precipitation trials. Using the selected stabilizing ligands diminished the average particle size from ca. 1,200 to 170–230 nm, reduced their dispersity, improved stability, and allowed reaching curcumin concentration of up to 1.4 mM in aqueous medium. Storage stability of the aqueous nanodispersions varied from 2 days to 2 weeks, depending on stabilizing ligand. Studying the effects of ionic strength and pH on size and *ζ*-potential of the particles suggested that electrostatic forces and hydrophobic interactions could be the major factors affecting their stability. The ligand-protected nanoparticles showed minimal inhibitory concentration of 400 or 500 µM toward *Escherichia coli.* We suggest that the presented screening approach may be useful for preparing nanoparticles of various poorly water-soluble bioactive materials.

## Introduction

Curcumin, a polyphenolic compound of plant origin and approved food additive [1] is a promising candidate for the development of natural antimicrobial nanomaterials. Curcumin (1*E*,6*E*)-1,7-bis(4-hydroxy-3-methoxyphenyl)-1,6-heptadiene-3,5-dione, is a major active constituent of turmeric, a spice prepared from dried ground rhizomes of *Curcuma longa (Zingiberaceae*) that has a long history of use in Asian medical tradition. Since first described by Schraufstätter and Bernt [2], the antimicrobial properties of curcumin have been demonstrated in a wide range of microorganisms [3–5] along with various health-beneficial effects [6]. Curcumin showed by far the highest antibacterial activity among the range of phenolic compounds tested by Rodov et al. [7]. There is ample evidence to support its potential use as a health promoting ingredient and as a natural preservative. However, poor aqueous solubility of curcumin creates an obstacle for delivering its bioactivity in food systems and pharmaceutical formulations. Similar to many other compounds of limited aqueous solubility, curcumin delivery may benefit from nanotechnological approaches.

The methods of nanoparticle production can be divided into two categories: top-down and bottom-up approaches. In the top-down methods larger units are mechanically broken to get micro or nanoparticles. High pressure homogenization [8] and wet milling [9] are examples of such methods applied to curcumin. On the other hand, the bottom-up approach relies on self-assembly of molecules into nanoscale aggregates. Liquid antisolvent precipitation (LASP) is one of the popular bottom-up methods that does not demand complicated and expensive equipment and is easy to scale up. The working principle behind this methodology is self-assembly of dissolved molecules into nanoparticles caused by a change in solvent properties. This is achieved when a solution of a poorly water-soluble compound in an organic solvent is mixed with an aqueous antisolvent. Supersaturation occurs in the solvent change resulting in nucleation and further particle growth due to the adsorption of the compound’s molecules. The LASP process is usually accompanied by mechanical and/or physicochemical interventions in order to control the particle growth and to prevent their agglomeration. Several versions of antisolvent techniques were employed previously for preparing curcumin nanoparticles using sonication [10, 11] and syringe pumping for agglomeration control [12].

**Fig. 1:**
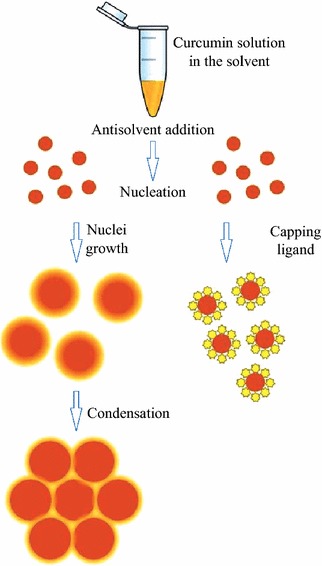
Scheme of preparing nanoparticles by antisolvent precipitation illustrating the effect of stabilizing capping ligands

The use of capping ligands illustrated in Fig. 1 is an additional way to control the growth of nanoparticles and to stabilize their dispersions [13]. Adsorption of stabilizing molecules on the surface of growing nanoparticles arrests their further growth by occupying the adsorption sites and inhibiting the incorporation of molecules into a particle, leading to a smaller particle size [14]. In addition, such ligands enhance electrostatic repulsion between particles and improve their thermodynamic stability [15]. To the best of our knowledge, the only report of using this approach with curcumin was published by Zheng et al. [10] who observed that coating with polyelectrolytes e.g., poly(allylamine hydrochloride) or protamine sulfate reduced the size and improved the stability of curcumin nanoparticles prepared by antisolvent crystallization. However, no attempts were made so far to optimize the procedure of preparing curcumin nanoparticles by choosing the most efficient stabilizing ligands.

The selection of proper stabilizer for a particular type of nanoparticles is not an easy task. Adsorption of a stabilizer and its efficacy are influenced by numerous factors such as interaction between functional groups of the stabilizer with the functional groups on the surface of the nanoparticle, interaction of a stabilizer with a surrounding medium, and surface energies of both nanoparticle and stabilizer [16]. As a consequence, rational and systematic use of stabilizing compounds is hampered by the need to perform a vast number of tedious trial and error experiments in the attempt to choose the suitable ones. High-throughput screening (HTS) tools have become an important part of drug discovery methodology, allowing parallel performance testing of numerous compounds. Application of this approach is expanding into new areas such as nanomaterial development. For example, the HTS approach has been applied recently by McDonald et al. [17] to the synthesis of nanoparticles of antimicrobial compound triclosan.

In this work, we present a new HTS methodology to optimize the preparation of stable ligand-coated nanoparticles of curcumin. Furthermore, the effects of various ligands on the stability of curcumin aqueous dispersions and on the properties and antimicrobial performance of curcumin nanoparticles are described.

## Work Approach

Reproduction of the antisolvent precipitation in a microplate is limited by mixing efficiency. As a working assumption, we hypothesized that promising ligands will exhibit their stabilizing capacity under non-optimal mixing conditions as well. The choice of candidate compounds for evaluation in the HTS assay was based on documented ability to stabilize nanoparticles reported in the literature (Table 1). The candidate compounds included low-molecular-weight substances (amino acids, polyamines, and phenolic compounds) and polymers. Both small phenolic molecules such as gallic acid, and bigger polyphenols such as epigallocatechin gallate and tannic acid (TA) were tested. Most of the polymers were represented by polyelectrolytes: polycationic amino acid homopolymers e.g., poly-l-lysine and poly-l-arginine, and polyanionic polysaccharides e.g., carboxymethyl cellulose. The group of nitrogen-containing polymers included polycations poly(allylamine hydrochloride), protamine sulfate, and Polyquaternium 10 (PQ-10) (quaternized hydroxyethylcellulose ethoxylate), as well as the only neutral polymer in this study, polyvinylpyrrolidone (PVP).

**Table 1:** Potential nanoparticle-stabilizing ligands tested in this study

Group		Reference	Substances tested
Low-molecular-weight substances	Amino acids	[18–20]	Glycine
			Proline
			Histidine
			Arginine
			Serine
			Methionine
			Phenylalanine
			l-DOPA
	Polyamines	[21]	Dopamine
			Spermine
			Spermidine
	Phenolic compounds	[22–24]	Gallic acid
			Epigallocatechin gallate
			Tannic acid
Polymers	Amino acid homopolymers	[25]	Polylysine (Mw 0.5–2 kDa)
			Polylysine (Mw 1–5 kDa)
			Polylysine (Mw 30–70 kDa)
			Polyarginine (Mw 5–15 kDa)
	*N*-containing polymers	[10, 26, 27]	Poly(allylamine hydrochloride)
			Protamine sulfate
			Polyquanternium 10
			Polyvinylpyrrolidone
	Anionic polysaccharides	[27–30]	Hydroxypropyl methylcellulose
			Carboxymethyl cellulose
			Dextrin
			Dextran sulfate
			Sodium alginate
			Polygalacturonic acid

## Experimental

All reagents used in this study were purchased from Sigma-Aldrich (St. Louis MO, USA). The deionized water (Milli-Q, Millipore Corporation, Billerica MA, USA) was used in the experiments.

### High-Throughput Screening

**Fig. 2:**
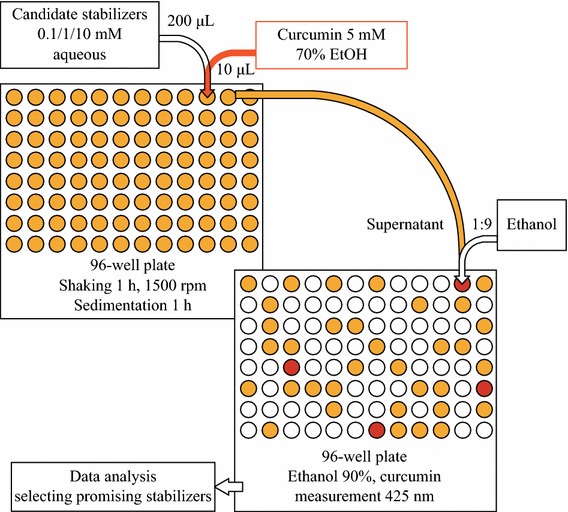
Scheme of the high-throughput screening assay

All the liquid handling steps in the HTS assays were performed using a Freedom Evo 75 robotic workstation equipped with on-deck Te-Shake orbital mixing platform and 8 plus 1 Access eight-channel liquid handling arm (Tecan Group Ltd, Durham, NC). The HTS assay scheme is presented in Fig. 2. Each candidate compound was used at three concentrations of 0.1, 1, and 10 mM. Molar concentrations for polymeric compounds were calculated based on molecular weights of the repeating unit. Water without a stabilizing compound was employed as a negative control and pollyallylamine and protamine sulfate known as good stabilizers of curcumin nanoparticles [10] as positive controls. The 10 μL of 5 mM curcumin solution in 70 % ethanol was added to the wells of the 96-well plate followed by addition of 200 μL volume of a stabilizing ligand candidate solution in water. The plates were shaken at 1,500 rpm for 1 h and left for an additional hour to allow sedimentation of big insoluble curcumin particles. The supernatant was transferred to another 96-well plate and diluted with ethanol to the final ethanol concentration of 90 %. The amount of solubilized curcumin was determined by measuring its absorbance at 425 nm with Synergy 2 Multi-Mode Microplate Reader (BioTek Instruments, Inc., Winooski, VT, USA) in comparison with calibration curve of curcumin in 90 % ethanol.

**Fig. 3:**
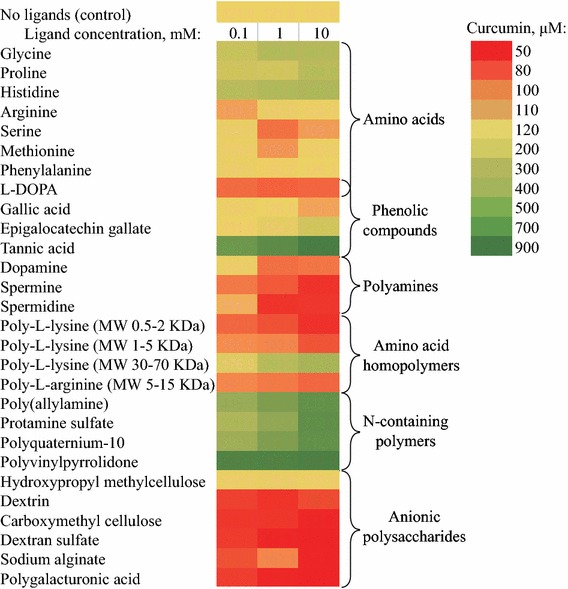
The results of the high-throughput screening assay represented as a heatmap

The assay was performed in triplicate. A Microsoft Office Excel spreadsheet was used to calculate means and standard deviations of the solubility data. The conditions at which water solubility of curcumin was at least two standard deviations higher than in negative control were regarded as a positive hit. The compounds were graded by their stabilizer efficacy according to the aqueous curcumin solubility reached (Fig. 3).

### Preparation of Curcumin Nanoparticles

Curcumin nanoparticles were prepared by ultrasound-assisted antisolvent precipitation using stabilizing ligands chosen from the HTS. The deionized water was used as an antisolvent. The syringe was filled with 19 mL of stabilizing ligand solution in antisolvent phase and added to 1 mL of the ethanolic curcumin solution (25 mM) under magnetic stirring at 1,000 rpm. The ligand concentrations were chosen on the basis of the HTS results. The mixture was sonicated for 20 min at 500 W, 20 kHz, using Q 500 sonicator (Qsonica LLC, Newtown, CT). The suspensions were left to stand for 1 h, allowing sedimentation of the non-dissolved curcumin aggregates; the supernatant nanoparticle dispersions were separated by decantation and used for further investigations.

### Scanning Electron Microscopy

The water dispersed nanoparticles were snap freezed in liquid nitrogen and freeze dried in a model FD5508 freeze dryer (ilShin Lab Co., Yangju, Gyeonggi, South Korea). Dry nanoparticle powders were spread over a carbon tape (Ted Pella, Inc., Redding CA, USA). The samples were introduced into a chamber of a field-emission environmental scanning electron microscope XL30 ESEM-FEG (Philips, Eindhoven, The Netherlands). The chamber was flooded at pressures between 0.4 and 0.7 Torr, and samples were observed using a secondary electron detector at 20–25 kV.

### Dynamic Light Scattering (DLS) and *ζ*-Potential Measurements

The measurements of nanoparticle size and *ζ*-potential were taken using a Zetasizer ZS Nano (Malvern Instruments, Malvern, UK) equipped with a He–Ne laser (*λ* = 633 nm) at 25 °C. For the size determination the light scattering was detected at an angle of 175°. DLS autocorrelation functions of the scattered light intensity were analyzed with DTS 5.0 software provided by the manufacturer, which allowed the measurement of the distribution of the scattered intensity versus the hydrodynamic diameters and determined the polydispersity index (PDI). For the measurement of *ζ*-potential, the electrophoretic mobility of the aggregates was determined by laser Doppler velocimetry, in disposable capillary cells. The *ζ*-potential values were calculated by the Smoluchowski approximation of Henry’s equation [31]. For the measurements of *ζ*-potential changes as a function of pH, the MPT-2 titrator accessory was used.

### Colloidal Stability

Colloidal stability was studied as a function of salt concentration using NaCl and CaCl_2_ as aggregating electrolytes. Particle aggregation was analyzed by dynamic light scattering using a Zetasizer Nano ZS system (Malvern Instruments). After experimental establishment of sample concentration appropriate for size measurement, the electrolyte solution of the desired ionic strength was added to 1 mL of diluted sample in a measurement cell and mixed by stirring. The measured diameter of the particles was plotted against time. The slopes of these plots (∂*d*/∂*t*) enabled determinations of the aggregation rate (*k*). The stability or Fuchs factor (*W*) was calculated from these data according to the equation

1 $$W\;=\;k_{r}/k_{s},$$

where the rate constant *k*_*r*_ corresponds to rapid coagulation kinetics, and the rate constant *k*_*s*_ corresponds to slow coagulation kinetics. Plotting the logarithm of *W* versus the logarithm of the salt concentration and locating the point where log*W* value reduced to zero gave the critical coagulation concentration (CCC), the minimal salt concentration for rapid aggregation of the colloidal system.

### Antimicrobial Activity

For microbiological experiments the ethanol was removed from the dispersions by evaporation under vacuum (Rotavapor R-124, Buchi, Flawil, Switzerland). The ethanol removal did not result in curcumin sedimentation. A starter culture was obtained by inoculating 25 mL of LB medium with a single colony of the *Escherichia coli* (ATCC 25922) and subsequent overnight growing at 37 °C with shaking at 200 rpm. This culture was diluted 1:400 and allowed to grow until it reached the mid-log phase (optical density of 0.4–0.5 at wavelength 600 nm). The cells were separated from the medium by centrifugation, washed with LB medium and resuspended in fresh threefold concentrated LB medium to optical density of 0.1 and then diluted with the same medium to obtain inoculum of 10^3^ colony forming units (CFU) per milliliter. The inoculums were further diluted 1:2 with aqueous nanoparticle dispersions and sterile water to obtain various concentrations of curcumin, while maintaining the same initial *E. coli* dilution and nutrient concentration. The bacterial suspensions were incubated for 20 h at 37 °C with shaking at 200 rpm. The experiments were run in triplicate and in addition to treatments (*E. coli* with curcumin nanoparticles) included positive controls (*E. coli* with nutrient medium with or without stabilizing ligands) and blanks (nutrient medium with nanoparticles without *E. coli*). The bacterial growth was registered by counting the CFU by the spread plate technique after 2, 4, 6, 8, and 20 h of growth. The 100 L aliquots of bacterial cultures were diluted serially in LB medium and grown on LB agar plates for 24 h at 37 °C. The colonies were counted and multiplied by dilution factor giving a number of viable CFU/mL. All platings were performed in triplicate. Bacterial growth curves were built by plotting log (*N/N*_*0*_) versus time, where N was the viable CFU mL^−1^ at a given time and *N*_*0*_ the viable CFU/mL at time zero. The concentration of curcumin at which the log (*N/N*_*0*_) value after 20 h was ≤0 was regarded as minimal inhibitory concentration (MIC).

## Results and Discussion

### High-Throughput Screening

The screening outcome is presented in Fig. 3. A remarkable ability to stabilize aqueous curcumin dispersions was demonstrated by the polyphenol TA while the smaller phenolics (gallic acid and epigallocatechin gallate) had poor stabilizing capacity. Among the amino acids only glycine, proline, and histidine produced moderately positive results. Negative results were shown by low-molecular-weight polyamines. Keeping in mind that TA was the biggest molecule among the non-polymeric substances tested, it may illustrate the relationship between stabilizer efficacy and number of binding sites in its molecule.

Cationic polymers polyallylamine and protamine sulfate had considerable stabilizing effect toward curcumin nanoparticles, in agreement with the previous report of Zheng et al. [10]. Similar or even higher activity was demonstrated by other nitrogen-containing polymers, a polycation Polyquaternium 10 (PQ-10), and especially a non-charged PVP. To the best of our knowledge, the potential of these materials for stabilizing curcumin nanoparticles hasn’t been reported previously.

All anionic polysaccharide-based polymers failed to provide stabilization and usually enhanced curcumin precipitation. One of the reasons for this effect might be electrostatic repulsion between nanoparticles and polymer molecules, both negatively charged. Noteworthy, the positively charged quaternarized polysaccharide derivative PQ-10 showed good stabilizing activity. In addition, nanoparticle aggregation could be promoted by the excluded volume effect of the polymers. Adding a solution of hydroxypropyl methyl cellulose (HPMC) to the curcumin caused flocculation, most probably due to cross linking of HPMC molecules by curcumin. The ability of HPMC to form complexes with phenolic compounds was exploited by Patel et al. [32] for preparing colloidal complexes.

Among the amino acid homopolymers, only high molecular weight poly-l-lysine (30–70 kDa) provided stabilization to the nanoparticles. The adsorption of polymer molecules on particle surface is described by tail–train–loop model [33]. The part of a polymer adsorbed on a particle surface is called train, parts of polymer with both ends in contact with particle surface are called loops, and the part which extends away from surface is called a tail. This last part of polymer chain provides thermodynamic protection for nanoparticle dispersion. It stands to reason, therefore, that shorter amino acid polymers failed to form such structures on nanoparticle surfaces and lead to aggregation by screening the particle surface charges.

### Nanoparticle Characterization

Based on the screening studies four representative stabilizing ligands were chosen for further investigations. The curcumin nanoparticles were produced in the presence of PVP, PQ-10, proline, and TA or without any stabilizers (control). Using ultrasound mixing resulted only in moderate improvement of curcumin solubility (1.5–3-fold compared with the results obtained in HTS experiments). On the other hand, with respect to dispersion stability, ultrasound provided a significant improvement. While the dispersions obtained in the positive hits of the screening experiments retained their stability only for relatively short time (5–10 h), the dispersions prepared with the chosen ligands under ultrasonic conditions were by far more stable. Proline-stabilized particles were stable for up to 2 days, TA-stabilized particles for a week, and particles stabilized with the polymers PVP and PQ-10 were stable for up to 2 weeks.

**Fig. 4:**
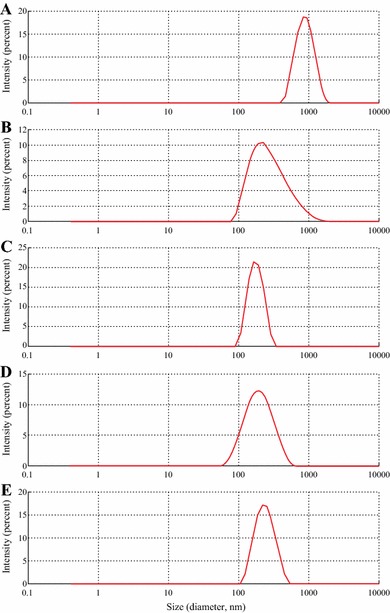
Size distribution of curcumin particles determined with dynamic light scattering (DLS). Stabilizing ligands: a particles without stabilizing ligands (control); **b** proline; **c** tannic acid; **d** polyvinylpyrrolidone; **e** polyquaternium 10

**Fig. 5:**
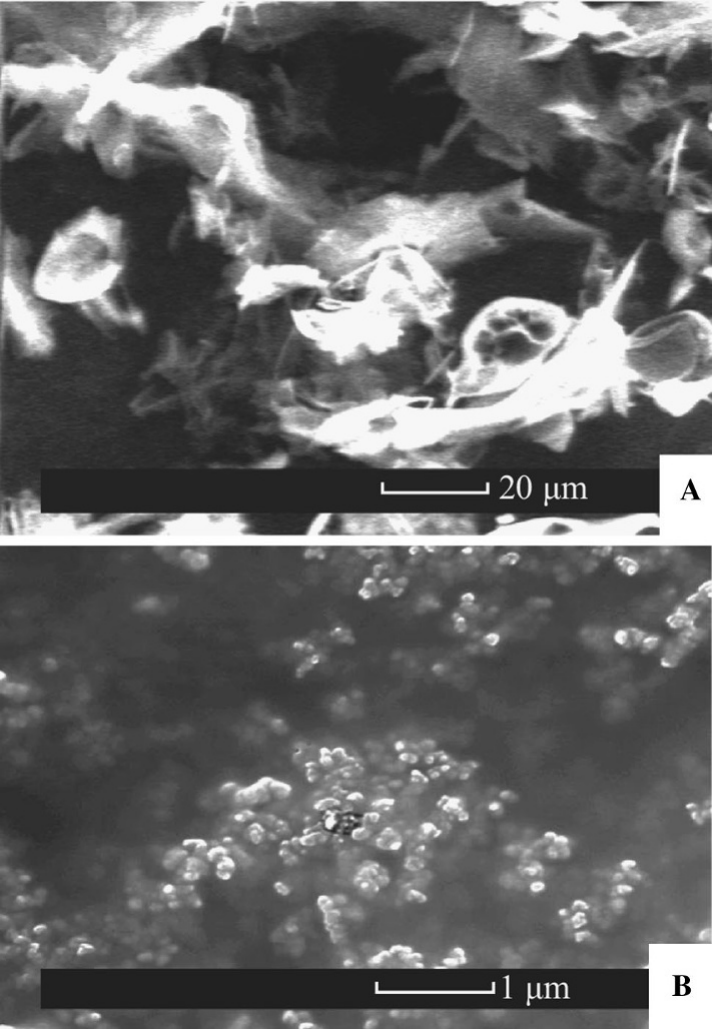
Environmental scanning electron microscopy images of the curcumin particles. **a** without stabilizing ligands (control), **b** stabilized with polyquaternium 10

The analysis by means of dynamic light scattering (Fig. 4) and environmental scanning electron microscopy (Fig. 5) had shown that particles prepared by antisolvent precipitation aided by capping ligands, had significantly smaller size (170–230 nm) and lower dispersity (PDI 0.08–0.2) compared to the particles prepared without the ligands (average size 1187 nm, PDI 0.5). The surface *ζ*-potentials measured by DLS are summarized in Table 2.

**Table 2:** Effect of selected stabilizing ligands on the characteristics of curcumin nanoparticles prepared by antisolvent precipitation

Ligand	Size (nm)	Polydispersity index (PDI)	*ζ*-potential (mV)	Curcumin (µM)^a^	Stability (days)^b^
None (control)	1187 ± 97	0.456	−18.2 ± 2.6	NA	NA
Proline	229 ± 62	0.207	−39.1 ± 2.9	860	2
Tannic acid	283 ± 71	0.274	−30.3 ± 4.1	1,400	7
Polyvinylpyrrolidone	175 ± 41	0.188	−2.2 ± 4.3	1,300	12
Polyquaternium-10	223 ± 54	0.081	+38.4 ± 3.2	1,200	16

^a^ Maximal curcumin concentration reached in stable aqueous dispersion

^b^ Storage duration of homogenous aqueous dispersion without visible separation

### Stability of the Nanoparticle Dispersions

**Fig. 6:**
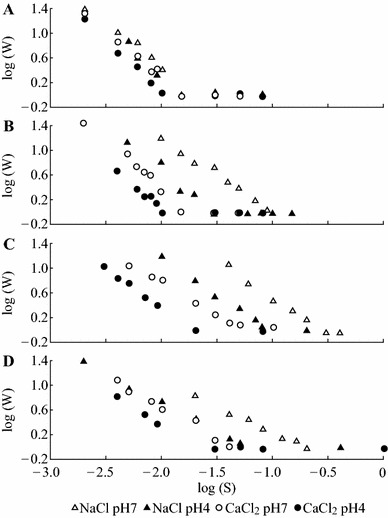
The effect of pH and ionic strength of the aggregating electrolytes on the stability (Fuchs factor) of the curcumin nanoparticles. Stabilizing ligands: **a** proline; **b** tannic acid; **c** polyvinylpyrrolidone; **d** polyquaternium 10

The stability of the dispersions was studied in the presence of NaCl and CaCl_2_ as aggregating electrolytes. The change of the particle size was examined at pH 4 and 7 as a function of time and salt concentration. The results of these tests allowed calculating the stability factors and the CCC for the nanoparticles protected with different ligands. Figure 6 shows the logarithm of the stability factor, log*W* plotted versus salt concentration for NaCl and CaCl_2_. The CCCs are summarized in Table 3.

**Table 3:** Effect of pH on critical coagulation concentration values (mM) for curcumin nanoparticles protected with different stabilizing ligands

Ligand	pH 7.0		pH 4.0	
	NaCl	CaCl_2_	NaCl	CaCl_2_
Proline	19	17	16	11
Tannic acid	113	19	30	11
Polyvinylpyrrolidone	265	53	93	18
Polyquaternium-10	179	45	61	18

According to the Derjaguin–Landau–Verwey–Overbeek (DLVO) theory [34, 35] electrostatic repulsion forces play a significant role in the stability of colloidal systems. *ζ*-potential provides an estimate of the electric potential at the boundary of the surrounding liquid layer attached to the particles and is widely used to predict suspension stability [36]. As a rule of thumb, higher *ζ*-potential is usually associated with greater stability to the suspension. However, in our trials proline-stabilized nanoparticles were only marginally stable at all experimental conditions despite their high *ζ*-potential (−40 mV). It is noteworthy that nanoparticles stabilized with PVP and PQ-10 had significantly higher CCC values and better stability than particles stabilized with low-molecular-weight ligands, proline, and TA, presumably due to significant input from thermodynamic stabilization mechanism.

**Fig. 7:**
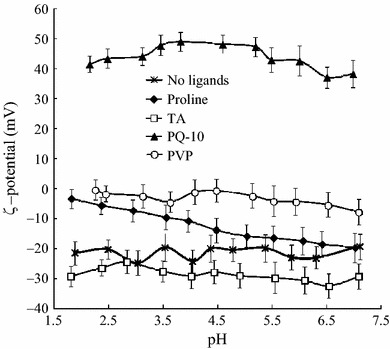
Change of the *ζ*-potential of curcumin nanoparticles as a function of pH. *Error bars* represent the standard deviation

The stability of PVP-, PQ-10-, and TA-protected nanoparticles was significantly reduced by acidic pH and high ionic strength as evident from the difference in CCC between NaCl and CaCl_2_. Nanoparticles protected with the TA had *ζ*-potential of −40 mV, sufficient for electrostatic stabilization in a solution and yet it provided high stabilization only at neutral pH and in the presence of NaCl. These discrepancies could be attributed either to changes in *ζ*-potential as a function of pH, or to significant impact of hydrophobic interactions known to be facilitated by high ionic strength and acidity. To provide experimental verification to either of these mechanisms, the *ζ*-potential was measured as a function of pH. The results presented in Fig. 7 show that with exception of proline-stabilized nanoparticles whose *ζ*-potential decreased almost linearly at acidic pH, *ζ*-potentials of the rest of nanoparticles showed no dependence on pH. This behavior was consistent with neutrality of PVP, pH-independent nature of the charges of the quaternary ammonium group of PQ-10, and weak acidity (pKa ~ 10) of TA. Therefore, it may be suggested that hydrophobic interactions rather than electrostatic screening were the main driving force behind the destabilization of PVP-, PQ-10-, and TA-protected curcumin nanodispersions under acidic pH and high ionic strength. The values of *ζ*-potential of the nanoparticles protected with proline were in a good agreement with the weak acidic character of their carboxylic groups, pointing to the possibility that the amino group was engaged in hydrogen bonding or in electrostatic interaction with nanoparticle surfaces.

### The Effect of Stabilizer Concentration

**Fig. 8:**
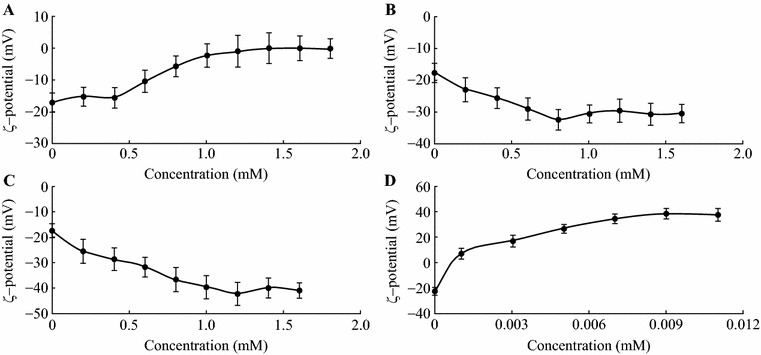
The effect of ligand concentrations on *ζ*-potential values of curcumin nanoparticles. Stabilizing ligands: **a** polyvinylpyrrolidone, **b** tannic acid, **c** proline, **d** polyquaternium 10. *Error bars* represent standard deviation

The effect of varying stabilizer concentration on resulting nanoparticle *ζ*-potential was studied for nanoparticles protected with proline, TA, PVP, and PQ-10 (Fig. 8). Unprotected curcumin particles showed a *ζ*-potential of about −18 mV. In case of proline- and TA-protected particles the increase of ligand concentration resulted in the shift of *ζ*-potential toward more negative values. In the particles protected with PVP and PQ-10, the *ζ*-potential values changed with increase of the ligand concentrations to almost 0 and +40 mV, respectively. The above behavior implies a Langmuir-like adsorption where the amount of the adsorbate on the surface increases with its concentration until complete coverage. Notably, cationic PQ-10 stabilized system reached the saturation at concentrations two orders of magnitude lower than the rest of the ligands, illustrating the importance of electrostatic interactions in ligand adsorption mechanism.

### Antimicrobial Activity

**Fig. 9:**
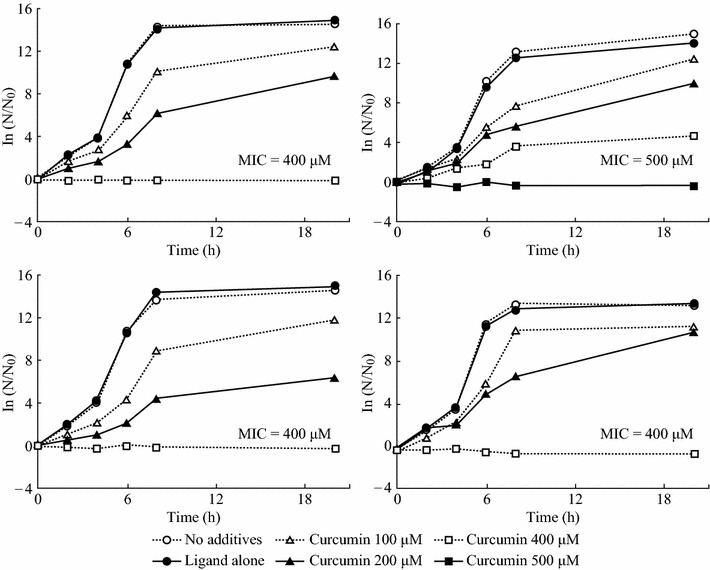
The effect of the nanodispersed curcumin concentration on the kinetics of *E. coli* growth and MIC values. Stabilizing ligands: **a** proline; **b** tannic acid; **c** polyvinylpyrrolidone; **d** polyquaternium 10

The effect of curcumin nanoparticles on growth of Gram-negative bacterium *E. coli* ATCC 25922 was examined in this study. Figure 9 shows the bacterial growth inhibition by curcumin nanoparticles stabilized with four different ligands. The nanoparticles showed control of bacterial growth at minimal curcumin concentrations of 500 μM (with TA) and 400 μM (with other ligands). These results are in agreement with the MIC value reported for *β*-methylcyclodextrin-encapsulated curcumin [7].

## Conclusions

This work presents a HTS of ligands to stabilize curcumin nanoparticles. Twenty-eight candidate compounds were tested utilizing antisolvent precipitation. Good stabilizing abilities were displayed by nitrogen-containing polymers, either cationic (polyallylamine, protamine sulfate, PQ-10, high molecular weight poly-l-lysine) or neutral (PVP), as well as by polyphenol TA, and some amino acids (histidine, glycine, and proline). Anionic polysaccharides and most low-molecular-weight compounds tested (polyamines, amino acids, and phenolic compounds) were inefficient and often enhanced the coagulation of curcumin.

The capacity of the HTS-selected molecules (proline, TA, PQ-10, and PVP) to stabilize curcumin nanoparticles was confirmed in the subsequent ultrasound-aided antisolvent precipitation tests. Stabilizing ligands significantly reduced the average particle size, improved stability, and allowed reaching curcumin concentration of up to 1.4 mM in aqueous medium. Studying the effects of ionic strength and pH on size and *ζ*-potential of the particles suggested that electrostatic forces and hydrophobic interactions could be the major factors affecting the particle stability. The ligand-protected curcumin nanoparticles showed antimicrobial activity toward Gram-negative *E. coli* ATCC 25922 with MIC values of 400–500 μM. The effect of stabilizing ligands on the antimicrobial activity of the particles deserves further investigation.

We believe that the presented HTS approach can be implemented also for preparing nanoparticles of other poorly water-soluble bioactive materials. Further research efforts in this project will be directed to testing the efficacy of the ligand-protected curcumin nanoparticles against other microbial species, elucidating the mode of antimicrobial action of the particles and their interaction with bacterial cell, and using the particles for preparing active antimicrobial food-contact surfaces.
